# Recurrent phage treatment of *Salmonella*-infected chickens transiently reshapes gut microbiota composition and function

**DOI:** 10.1186/s13567-025-01708-4

**Published:** 2026-03-14

**Authors:** Lorna Agapé, Florent Kempf, Pierrette Menanteau, Madeline Morinet, Catherine Schouler, Olivier Boulesteix, Mickaël Riou, Isabelle Virlogeux-Payant, Philippe Velge

**Affiliations:** 1 INRAE, UMR ISP, Université de Tours, 37380 Nouzilly, France; 2 INRAE, UE-1277 Plateforme d’Infectiologie Expérimentale (PFIE), 37380 Nouzilly, France

**Keywords:** Bacteriophage, *Salmonella*, gut microbiota, functional property

## Abstract

Bacteriophages (phages) are viruses that target bacteria and hold great promise as therapeutics against multidrug-resistant bacteria, pathogen infections, and for microbiota engineering. Despite their potential, the effect of phage therapy on gut microbiota remains insufficiently explored. This study investigated the impacts of recurrent phage administration on the gut microbiota of chickens challenged with *Salmonella* Enteritidis. Eighty chicks were infected at 7 days of age and either treated with a cocktail of six lytic phages of *Salmonella* via drinking water for 21 days before and after infection, or left untreated. Surprisingly, continuous high-dose phage treatment led to higher *Salmonella* colonization levels than short-term pre-infection exposure. Through fecal and cecal microbiota analysis, we found that phage treatment significantly influenced alpha and beta diversities as well as microbiota development, with the most significant changes occurring prior to *Salmonella* infection, and gradually diminishing over time. Furthermore, phage treatment transiently altered predicted microbiota functions, promoting an oxygen-utilizing microbial community that may have counteracted the protective effects of phages. These findings demonstrate that phages can induce time-dependent modifications in both the composition and functional profile of the gut microbiota, emphasizing the importance of considering these dynamic interactions when developing phage-based strategies against enteric pathogens.

## Introduction

Phages are viruses specifically targeting bacteria. Infection by lytic phages leads to the death of their host. They may be subsequently used to control bacterial infection, as an alternative to antibiotics [1], or are proposed as targeted therapeutics against multidrug-resistant bacteria [2]. They present multiple advantages: low toxicity, the “auto dosing” property (the fact that phages are able to increase their numbers in the presence of their host), a lower potential than antibiotics to induce long-term resistance due to phage adaptation, the lack of cross-resistance with antibiotics, a rapid discovery process, their versatility of use, and the clearance of biofilms [3]. The introduction of phages into the ecosystem of the host can induce some changes in the gut microbiota composition, a phenomenon that is often neglected. Some studies have reported that phages induced limited changes within the gut microbiota, even in the case that they successfully reduced the host bacterial load. For example, Richards et al. showed that a phage cocktail specifically targeting *Campylobacter jejuni* reduced the bacterial load without inducing major changes in the gut microbiota composition [4]. Other studies demonstrated that, depending on the time of phage administration during the life of the chickens, phages targeting *Salmonella* may affect the gut microbiota, potentially leading to beneficial effects on gut health [5]. The change induced may also increase favorable bacteria, such as short-chain fatty acids (SCFA) producers [6]. Lastly, in the context of *Salmonella*-infected chickens, phages may enhance performance traits and reduce changes in the gut microbiota induced by the bacterial pathogen [7]. All these results suggest either negligible or favorable effects of phages on the gut microbiota. However, other studies have shown that the application of *Salmonella* phages under field conditions modulated the cecal microbiome and metabolome profiles in broilers [8], owing to direct bacterial lysis effects or indirect cascade effects [9, 10]. Indeed, different bacteria are interconnected in the microbiota and treatment with phages not only induced the elimination of susceptible bacteria but also induced cascading effects on other species through the interactions between bacteria [9, 11]. If the precise modulation of microbiota via phage therapy shows potential for treating microbiota-associated diseases, other studies have shown that phages targeting protective members of the microbiota may increase the risk for *Salmonella* infection [12]. It therefore seems important to test the effect of phage administration on the microbiota.

*Salmonella* is one of the main causes of foodborne diseases and represents a major public health and economic burden [13]. For example, salmonellosis is the second most commonly reported bacterial foodborne gastrointestinal infection in humans after campylobacteriosis in Europe and the USA [14]. Among all *Salmonella* serovars, Enteritidis is predominant in Europe and the USA: it is associated with 79.7% of *Salmonella* outbreaks. These foodborne outbreaks are mainly caused by the consumption of poultry products, including eggs and poultry meat [13]. In chickens, *Salmonella* typically induces an asymptomatic carrier state that can prevent effective diagnosis, and is associated with a super-shedder phenotype [15]. This super-shedding state is of crucial importance for *Salmonella* epidemics as 20% of the most excreting animals are responsible for 80% of cross-contamination [16]. Prevention of the infection is therefore a control measure of great relevance against *Salmonella* in poultry. From this point of view, phage administration is a promising avenue for preventive strategies. By comparison, vaccines can indeed be costly and ineffective in young animals [17]. In line with this, while antibiotics have been widely used as growth promoters and in preventive measures, their use has led to the emergence of multidrug-resistant bacteria that pose a threat to human health. For this reason, they are currently banned in Europe to control salmonellosis in poultry.

The phage cocktail used in the present study contains six lytic phages targeting *Salmonella* and has been proved to be effective against *S.* Enteritidis infection in chicks when used preventively [18]. However, this phage cocktail was less effective when used before and during infection. This lower efficacy was partly linked to the adaptation of *Salmonella* to recurrent phage inoculation [19]. However, it could also be linked to a modification of the intestinal microbiota, which plays a major role in *Salmonella* colonization [20, 21]. This article therefore focuses on the impact of recurrent phage administration (i.e., before and after infection) on the composition of the gut microbiota and its predicted metabolic functions. This was done on the basis of an in vivo assay comparing the composition of fecal and cecal microbiota from infected chickens receiving or not receiving the phage cocktail.

## Materials and methods

### Ethics statement

The in vivo experimental infection was performed at the PFIE (UE-1277 PFIE, INRAE Centre de Recherche Val de Loire, France). The experiment was carried out in strict accordance with French legislation. All animal care and use adhered to French animal welfare laws. The protocol for this study was approved by the Loire Valley ethical review board (CEEA VdL, committee number 19) and the French Ministry of Education, Higher Education and Research (Ministère de l’Education Nationale, de l’Enseignement Supérieur et de la Recherche) under the protocol no. APAFIS#24688–202009081211981 v1. The principles of reduction, replacement, and refinement were implemented in the experiment.

### Bacterial strain and phages used in this study

A spontaneous streptomycin-resistant variant of the *Salmonella* Enteritidis LA5 strain (SELA5-775), originally isolated from broiler chicken and also resistant to nalidixic acid, was used in this study [22, 23]. For liquid cultures, bacteria, preserved in 50% (v/v) glycerol, were routinely cultivated in lysogeny broth (LB, Miller formula) and grown overnight at 37 °C at 180 rpm.

A phage cocktail preparation, supplied by a commercial company specializing in the development and commercialization of phage therapeutics, was used in this study. It contained six individual bacteriophages isolated from environmental water, designated as SalE_1, SalE_2, SalE_3, SalE_4, SalE_5, and SalE_6, and has been previously described [18]. The six phages have different spectrums of action on *Salmonella* strains, belong to different genera, and have different genome sizes. Five of them (SalE_2, SalE_3, SalE_4, SalE_5, and SalE_6) have lytic activity against the *Salmonella* Enteritidis LA5 and the SELA5-775 strains.

### Host range determination

Phage host range determination was performed on a panel of 70 strains using the spot assays conducted on a double agar overlay [24]. Among the 70 strains, 8 *Salmonella* strains and 62 avian commensal *Escherichia coli* strains of INRAE collection (with a BEN number) recovered from adult poultry gut were tested. Briefly, phages were serially diluted tenfold in Dulbecco phosphate-buffered saline (DPBS) (Thermo Fisher Scientific, USA) and spotted onto a double agar overlay containing lawns of the targeted strain. The agar overlay consisted of molten 0.5% (w/v) LB agarose supplemented with 10 mM MgSO_4_, 1 mM CaCl_2_, and 30 µM 2,3,5-triphenyltetrazolium chloride, which was poured onto 15% (w/v) LB agar plates, as previously described [18]. Plates were then visually examined to assess the extent of the lysis zone, including cases where no visible lysis was observed.

### Experimental design

**Figure 1:**
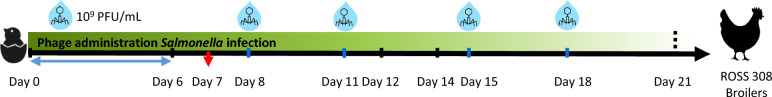
**Schematic experimental design.** Broiler chicks were separated in two groups of 40 birds and challenged with *Salmonella* Enteritidis SELA5-775 at 7 days of age (indicated by a red arrow). The 40 chicks of the Φ + SE group received a phage cocktail administrated orally via drinking water (1 × 10^9^ PFU/mL) during the first 6 days of life. Following infection, fresh phage administrations were provided in the drinking water at 8, 11, 15, and 18 days of age (indicated in blue). To monitor *Salmonella* colonization, eight chicks from each group were randomly removed, euthanized, and sampled at 6, 8, 12, 14, and 21 days of age for the collection of fecal and cecal samples.

The experimental design has been previously described [19] and all the cecal and fecal samples used for microbiota analysis were obtained from that study. Briefly, two groups of 40 broiler (tested negative for *Salmonella*) chicks (namely the “SE” and “Φ + SE” groups) were reared in the same confined room to avoid the batch effect owing to a different environment but were housed in separate pens on straw litters to avoid microbiota exchanges. All chicks were challenged by oral gavage with *Salmonella* Enteritidis SELA5-775 at 5 × 10^4^ CFU/chick at 7 days of age. Chicks of the Φ + SE group were also orally inoculated by the phage cocktail present in drinking water (1 × 10^9^ PFU/mL) during the first 6 days of life, and then after infection at 8, 11, 15, and 18 days of age (Figure 1), for studying the effect of a recurrent administration.

Chicks of the SE group were tested for the absence of the phages used in the cocktail. At 6 (i.e., before *Salmonella* inoculation), 8, 12, 14, and 21 (i.e., after *Salmonella* inoculation) days of age, all chicks were weighed and eight chicks were removed from each of the SE and Φ + SE groups for the collection of fecal samples before euthanasia, which were then weighed and stored at −80 °C for further metabarcoding analyses. For this, the chicks were kept separate until they defecated. Then, these eight chicks were euthanized and their cecal contents were collected, weighed, and stored at −80 °C for subsequent microbiota analyses. No significant difference in weight was detected between the two groups of animals. Levels of *Salmonella* and phages were measured as previously described [18].

### DNA extraction and 16S rRNA gene sequencing

The sample collection yielded a total of 145 fecal and cecal samples used for metabarcoding analyses (39 cecal and 38 fecal samples in the Φ + SE group and 40 cecal and 28 fecal samples in the SE group). DNA was extracted using the NucleoMag^®^ DNA Microbiome kit following the manufacturer’s instructions (Macherey–Nagel, Hoerdt, France) and automated via the Microlab Nimbus 100 robot (NIMBUS4, Hamilton, Rungis, France) except that a mechanical lysis step was added with the Precellys Evolution instrument with activated Cryolys (Bertin Technologies, Aix-en-Provence, France) for 6 × 90 s at 9000 rpm and 4 °C. Nanodrop One (Thermo Scientific) was used to quantity DNA samples. In total, 15 samples were excluded from further analyses owing to inadequate DNA concentrations after DNA extraction. The V3–V4 regions of the bacterial 16S rRNA gene were PCR amplified using the following forward and reverse primers, respectively: 5′-TCGTCGGCAGCGTCAGATGTGTATAAGAGACAG-[CCTACGGGNGGCWGCAG]−3′ and 5′-GTCTCGTGGGCTCGGAGATGTGTATAAGAGACAG-[GACTACHVGGGTATCTAATCC]−3′. Then, genomic DNA was sequenced on an Illumina MiSeq by Genomer platform (EMBRC France partner, Roscoff, France) using paired end 2 × 300 bp cycles.

### 16S rRNA gene sequence analysis

Bioinformatic 16S rRNA gene sequence analyses were performed using the FROGS analysis pipeline [25]. The read assembly yielded a total of 3 728 592 16S rRNA gene sequences. They were clustered into 750 096 clusters using Swarm with a distance of 1 [26]. After removal of chimeric reads (detected using VSearch [27]) and low-quality reads, 460 463 clusters (2 915 084 sequences) were kept. Following the exclusion of very rare clusters (relative abundance < 0.005% of all sequences [28]) and sequences matching the phiX contaminant database [25], 559 operational taxonomic units (OTUs) (representing a total of 2 354 684 sequences) were retained for further analysis. These OTUs were assigned to the lowest taxonomic category using a BLAST comparison with the SILVA 16S 132 database [29, 30].

### Functional metagenomic predictions

Functional gene families and MetaCyc pathways were predicted from taxonomic abundance data using PICRUSt2 [31]. Predicted MetaCyc pathways were then aggregated at the super-pathway level on the basis of the MetaCyc database [32].

### Statistical analyses

Most statistical analyses were performed using R packages. All samples underwent rarefaction for normalization, and relative abundance comparisons were performed using the R package phyloseq (v1.40.0 [33]). α-Diversity was measured using Chao1 and Shannon indices and computed using the “estimate_richness” function of phyloseq. Analysis of variance (ANOVA) tests were performed to determine α-diversity changes between all the groups, and Welch’s *t*-tests were used to test for differences in mean α-diversity between groups. β-Diversities were computed using the “ordinate” function of phyloseq and the Bray–Curtis index. Permutational multivariate analysis of variance analysis (PERMANOVA, 9999 permutations) was used to compare mean β-diversity indices among the groups. This was done using the “adonis2” function of the R package Vegan [34]. Differential abundances between groups at the OTU level were assessed using DESeq2 package (v1.36.0) [35] (*p*-values < 0.01 after correction using the Benjamini–Hochberg’s method). Only OTUs or pathways presenting a log_2_ fold change higher than 2 or lower than −2 were kept. Tests of differential abundances at the family and genus level were made using Welch’s *t*-tests corrected using the Benjamini–Hochberg’s method. This was done using STAMP v2.1.3 [36].

## Results

### The phages used in the in vivo trial have a lytic activity on *Salmonella* and some commensal *E. coli* strains

**Figure 2:**
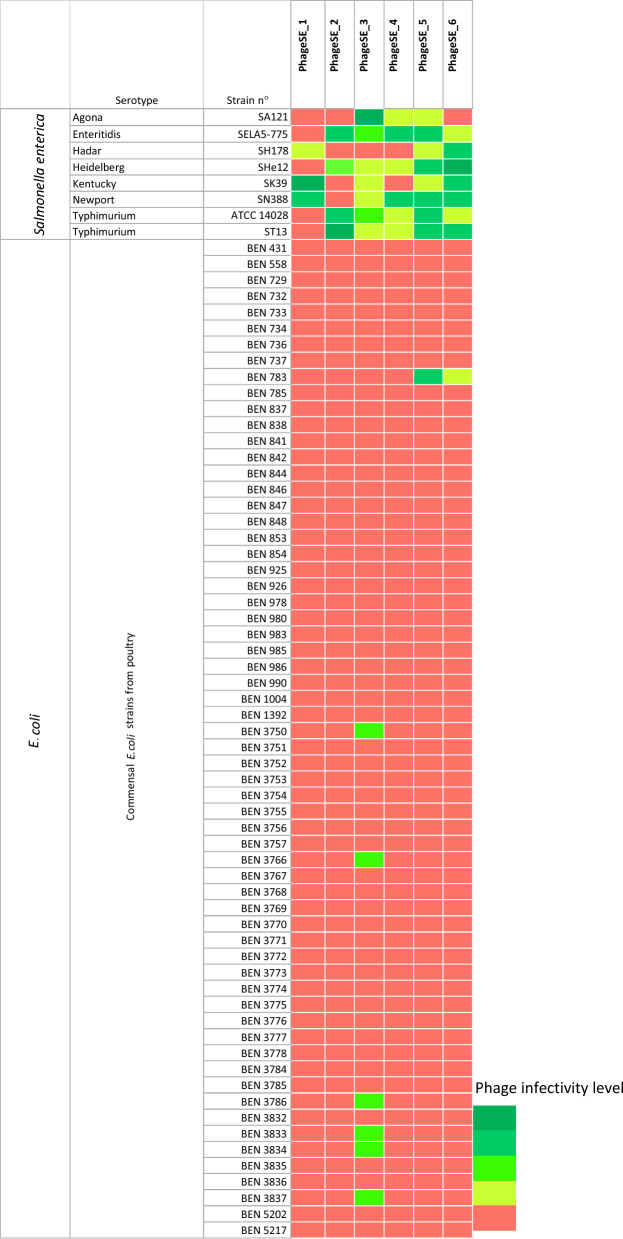
**Host range of the phages used**. Host range of the six phages of the cocktail represented by a heatmap of the infectivity profile of each single phage. Phage lysis activity was determined by a spot test assay on eight *Salmonella* strains, representing different serotypes, and 62 commensal *E. coli* strains recovered from poultry gut. Green areas represent complete and clear lysis. Red areas represent an absence of lysis.

The lytic ability of the phages was tested on various strains to determine, in vitro, the host range of the cocktail (Figure 2). Among the *Salmonella* strains analyzed, the cocktail showed a relatively broad host range, infecting all eight *Salmonella* strains tested with at least three phages from the cocktail active against them. As the phage cocktail targets different *Salmonella* serotypes, the potential effect of the phages on other *Enterobacteriaceae* such as *E. coli* was also investigated. A panel of 62 nonpathogenic *E. coli* strains recovered from chicken intestines was tested, and 7 of them were found to be sensitive to some of the phages in the cocktail. SalE_3 was able to lyse six of them, and SalE_5 and SalE_6 were able to lyse the BEN 783 strain. These results reinforced the need to check the effect of the phage cocktail on cecal and fecal microbiota. Phages could indeed have a direct effect by eliminating *Salmonella*, but they could also induce cascade effects on other species through interactions with commensal *E. coli* [9, 11].

### The gut microbial composition varied according to the age and the organ (feces versus ceca)

**Figure 3:**
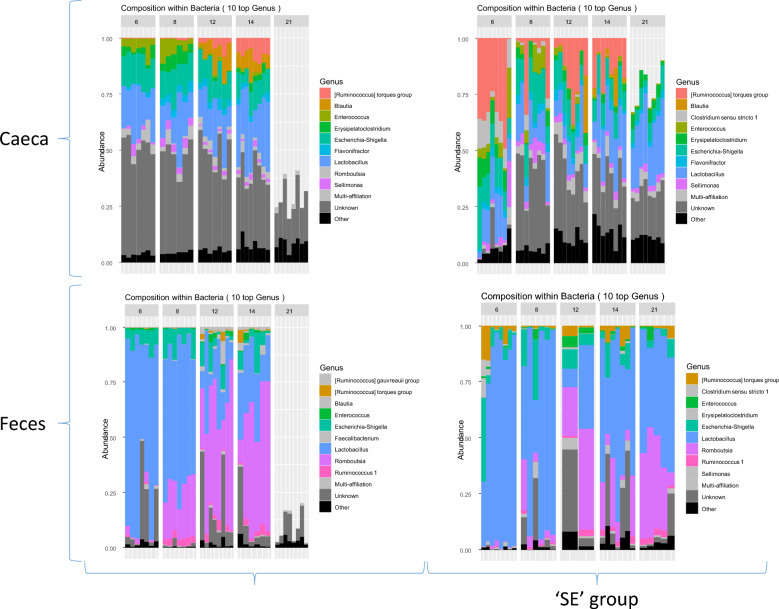
**Relative abundance of bacteria for the Φ + SE and SE groups**. Taxonomic bar plots showing the relative abundance of bacteria at the genus taxonomic level from chicken cecal and fecal samples collected at each time point for the Φ + SE and SE groups.

**Figure 4:**
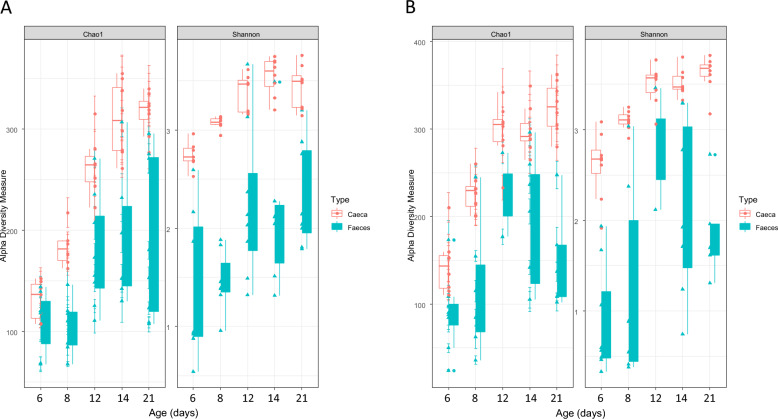
**Diversity of cecal and fecal microbiota for the Φ + SE and SE groups**. α-Diversities at each time point were measured with the Chao1 and Shannon indices and compared between cecal and fecal samples for the Φ + SE (**A**) and SE (**B**) groups.

We first assessed the differences and similarities in gut microbial compositions between cecal and fecal samples to investigate correlations between the cecal and fecal microbiota of the same animal and find out whether one organ can be a proxy for the other, or whether the impact of phages on the structure of both microbial communities needs to be studied. The taxonomic composition presented in Figure 3 highlights major differences between the cecal and fecal microbiota. For example, the *Ruminococcus torques* group, which is a strictly anaerobic bacterium known for producing many short chain fatty acids (SCFA), was consistently more abundant in cecal samples than in fecal samples across all time points (16% and 3.0% in the SE groups, respectively). By contrast, in the fecal samples, we observed higher levels of *Lactobacillus* than in ceca (61.8% and 17.3% in the SE groups, respectively) as well as a greater abundance of *Romboutsia* (13.4% and 1.4%, respectively). Moreover, as previously described, this microbiota evolved with age. For example, very few *Romboutsia* were detected at 6 days of age (DoA), a percentage that increased over time to reach 49% at 21 DoA in feces. These differences between cecal and fecal samples were confirmed by computing α-diversity indices (i.e., Chao1 and Shannon) at each time point. Figure 4 clearly shows that α-diversity increased with time and remained higher in the cecal samples than in fecal samples (*p*-values ranging from 0.006 to < 0.001). This was an expected result showing that chicks developed a more complex gut microbiota over time, with patterns of α-diversity reflecting colonization of the gut by successive bacteria. For example, cecal α-diversity increased sharply between 6 and 8 DoA and between 8 and 12 DoA (Table 1) whereas fecal α-diversity increased less, as only a significant increase was observed between 8 and 12 DoA in the Φ + SE group (Table 1). The differences between cecal and fecal samples were also reflected by dramatic differences in β-diversity measures using the Bray–Curtis index. At each time point, for each group (Φ + SE and SE), tests revealed different patterns of β-diversity in the fecal and cecal samples (*p*-values ranging from *p* = 0.023 to *p* < 0.001). Overall, these results indicated striking differences in quantitative and qualitative aspects of gut microbial composition between fecal and cecal samples. These data clearly confirmed the importance of measuring, kinetically, the effect of phages on both the fecal and cecal samples.

**Table 1:** ***p*-values of the Welch’s** ***t*-tests comparing the α-diversities assessed using Shannon and Chao1 indices for each temporal shift**

	Φ + SE group	SE group
*Ceca*		
Chao1		
6 → 8 days of age	**< 0.001**	**< 0.001**
8 → 12 days of age	**< 0.001**	**< 0.001**
12 → 14 days of age	**0.016**	0.960
14 → 21 days of age	0.687	0.081
Shannon		
6 → 8 days of age	**< 0.001**	**0.007**
8 → 12 days of age	**0.004**	**< 0.001**
12 → 14 days of age	0.115	0.776
14 → 21 days of age	0.3321	0.280
*Feces*		
Chao1		
6 → 8 days of age	0.784	0.459
8 → 12 days of age	**0.007**	0.201
12 → 14 days of age	0.662	0.631
14 → 21 days of age	0.916	0.326
Shannon		
6 → 8 days of age	0.889	0.449
8 → 12 days of age	**0.029**	0.199
12 → 14 days of age	0.770	0.503
14 → 21 days of age	0.596	0.548

Significant *p*-values are in bold.

### Recurrent phage administration has a small but significant impact on microbial excretion

When comparing the evolution of microbiota composition in fecal samples over time between the Φ + SE and SE groups, we observed relatively minor yet significant differences. As shown in Figure 3, under our experimental conditions, both groups exhibited a fecal microbiota dominated by *Lactobacillus* in the first days of life, which gradually declined and was replaced by *Romboutsia*. However, significant differences emerged between the two groups. Although α-diversities, assessed using the Shannon and Chao1 indices, increased over time (Figure 4), no significant difference were observed between two consecutive time points in the SE group, and only minor differences were detected in the Φ + SE group (Table 1). Furthermore, the Bray–Curtis β-diversity index revealed significant differences between the microbiota of the two groups at 6 and 14 DoA (Table 2). Thus, the dynamics of fecal bacterial compositions slightly differed between the two groups. In the Φ + SE group, microbiota composition significantly shifted between 6 and 8 DoA and again between 8 and 12 DoA, whereas no significant changes were observed in the SE group during these periods (Table 3).

**Table 2:** ***p*-values of the tests comparing the β-diversities between the Φ + SE and SE groups assessed using the Bray–Curtis index**

	Ceca	Feces
6 days of age	**< 0.001**	**0.043**
8 days of age	**0.036**	0.051
12 days of age	**< 0.001**	0.739
14 days of age	**< 0.001**	**0.005**
21 days of age	**0.030**	0.130

Significant *p*-values are in bold.

**Table 3:** ***p*-values of the tests comparing the β-diversities assessed using the Bray–Curtis index for each temporal shift**

	Φ + SE group	SE group
*Ceca*		
6 → 8 days of age	**0.004**	**0.001**
8 → 12 days of age	**< 0.001**	**0.001**
12 → 14 days of age	**0.006**	0.098
14 → 21 days of age	**< 0.001**	**0.002**
*Feces*		
6 → 8 days of age	**< 0.001**	0.226
8 → 12 days of age	**< 0.001**	0.076
12 → 14 days of age	0.599	0.198
14 → 21 days of age	0.121	0.200

Significant *p*-values are in bold.

**Figure 5:**
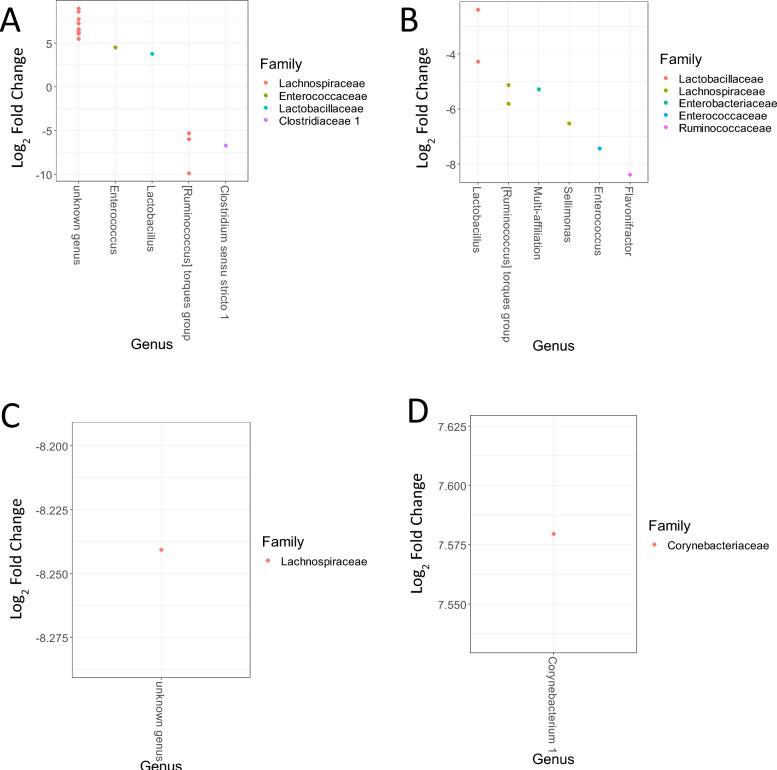
**OTUs presenting differential abundances in the fecal samples between the SE and Φ + SE groups**. Analysis of OTUs was performed in the fecal samples at 6 days of age (**A**), 8 days of age (**B**), 14 days of age (**C**), and 21 days of age (**D**). Each dot represents one OTU. For each OTU, the genus is reported on the *x*-axis. The families are represented using different colors. Positive log_2_ fold changes correspond to OTUs enriched in the Φ + SE group. Reciprocally, negative log_2_ fold changes correspond to OTUs enriched in the SE group.

We next investigated the taxa with differential abundances between the two conditions in fecal samples, focusing on those potentially influenced directly or indirectly by phage treatment (Figure 5). Focusing on taxa showing at least 1% abundance in one of the groups, we observed that only a few taxa presented differential abundances between the Φ + SE and SE groups at 6 and 8 DoA in the fecal samples. In the latter, only one taxon was different at 14 and 21 DoA and no significant difference was observed at 28 DoA. Interestingly, at 6 DoA, i.e., just after the first stop of phage inoculation and before *Salmonella* inoculation, facultative anaerobes (*Enterococcus* and *Lactobacillus*) were more abundant in the Φ + SE group while strict, SCFA-producing anaerobes were more abundant in the SE group. This result suggested that phage treatment altered the ecological niche of the fecal microbiota even though we also detected enrichment of strictly anaerobic bacteria (from the *Lachnospiraceae* family) in the Φ + SE group.

### Recurrent phage administration has a significant and important impact on cecal microbiota

Figure 3 illustrates marked differences in the most abundant cecal bacterial populations between phage-treated and untreated chicks, particularly at 6 DoA. Notably, and consistent with observations in fecal samples, the *Ruminococcus torques* group was highly abundant in the SE group but absent in the Φ + SE group at both 6 and 8 DoA. Furthermore, compared with feces, the dynamics of the cecal microbial compositions were important but similar between the Φ + SE and SE group, except between 12 and 14 DoA where β-diversities between two consecutive time points did not change in the SE group, whereas they changed significantly in the Φ + SE group (Table 3). These differences showed that over the course of the experiment, the chicks developed a more complex cecal microbiota, with patterns of α-diversity reflecting colonization of the gut by successive bacteria. However, significant differences were observed in β-diversities between the Φ + SE and SE groups at all time points (Table 2), suggesting that phage administration has an impact on the composition and evolution of cecal microbiota.

**Figure 6:**
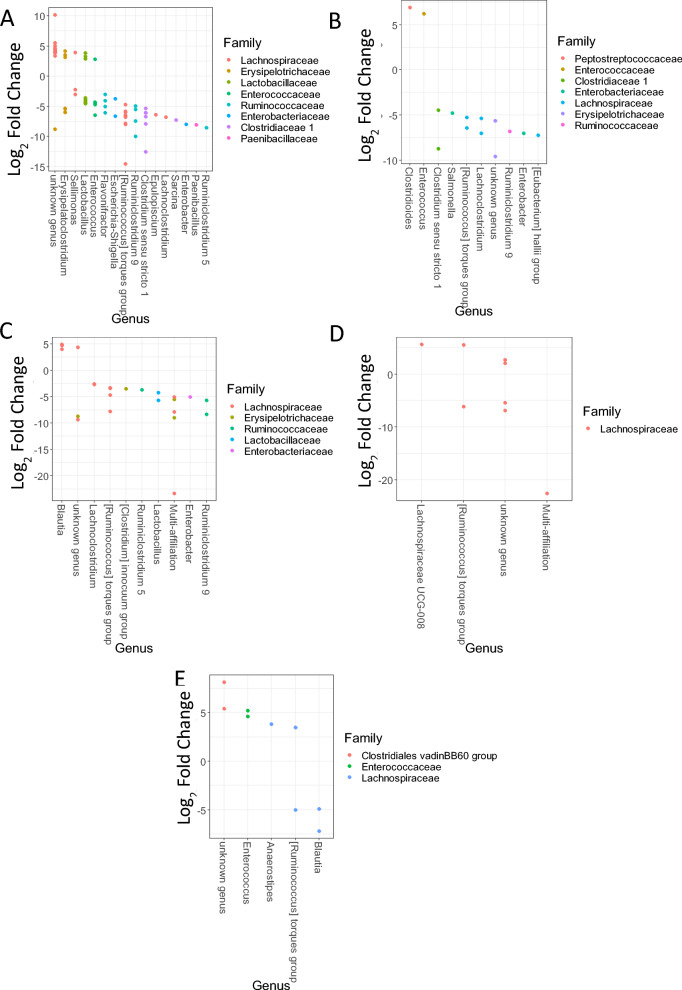
**OTUs presenting differential abundances in the cecal samples between the SE and Φ + SE groups**. Analysis of OTUs was performed in the cecal samples at 6 days of age (**A**), 8 days of age (**B**), 12 days of age (**C**), 14 days of age (**D**), and 21 days of age (**E**). Each dot represents one OTU. For each OTU, the genus is reported on the *x*-axis. The families are represented using different colors. Positive log_2_ fold changes correspond to OTUs enriched in the Φ + SE group; reciprocally, negative log_2_ fold changes correspond to OTUs enriched in the SE group.

When we investigated the taxa in the ceca that could explain these differences in β-diversity, we observed that many bacterial genera were significantly different between the two conditions, particularly at 6 DoA (Figure 6). Focusing on taxa with at least 1% abundance in either group, we identified 126 OTUs that were differentially abundant between the Φ + SE and SE groups across all time points. Given that phages cannot directly infect all these bacteria, this finding suggests that phage treatment had a substantial indirect effect on the cecal microbiota.

At 6 DoA, phage treatment led to an increased abundance of certain OTUs from the *Lachnospiraceae*, *Erysipelotrichaceae*, *Lactobacillaceae*, and *Enterobacteriaceae* families. However, this did not appear to substantially modify the ecological niche, as other OTUs within the same families were also enriched in the Φ + SE group, suggesting a functional or compositional compensation within the microbial community.

In contrast, numerous genera from several families were enriched in the SE group, including OTUs from the *Enterobacteriaceae*, which were depleted in the Φ + SE group (or enriched in the SE group) at 6, 8, and 12 DoA. Interestingly, one OTU identified as *Salmonella* was more abundant in the SE group than in the Φ + SE group, suggesting a direct effect of the phages. Nevertheless, these OTUs from *Enterobacteriaceae* corresponded to a low number of sequences (inferior to 400 in the SE group). Overall, both direct and indirect effects of phage treatment appeared to diminish over time, as the number of differentially abundant OTUs decrease toward the end of the experiment.

### Recurrent phage administration functionally modulated the cecal gut microbiota

**Figure 7:**
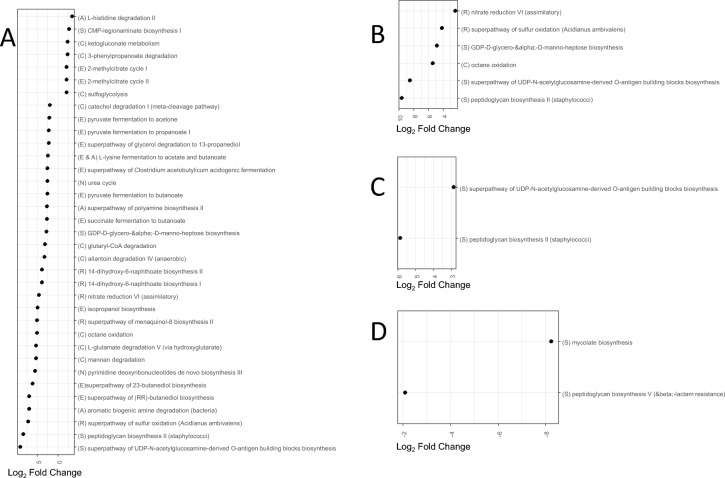
**Metabolic pathways presenting differential enrichment in the cecal samples**. Functional gene families and MetaCyc pathways were predicted on the basis of the taxa abundances using PICRUSt2 on microbiota at days 6 (**A**), 8 (**B**), 12 (**C**), and 21 (**D**). No differentially enriched pathways were observed at 14 days of age. Positive values of log_2_ fold changes correspond to pathways enriched in the SE group; reciprocally, negative values of log_2_ fold changes correspond to pathways enriched in the Φ + SE group.

**Figure 8:**
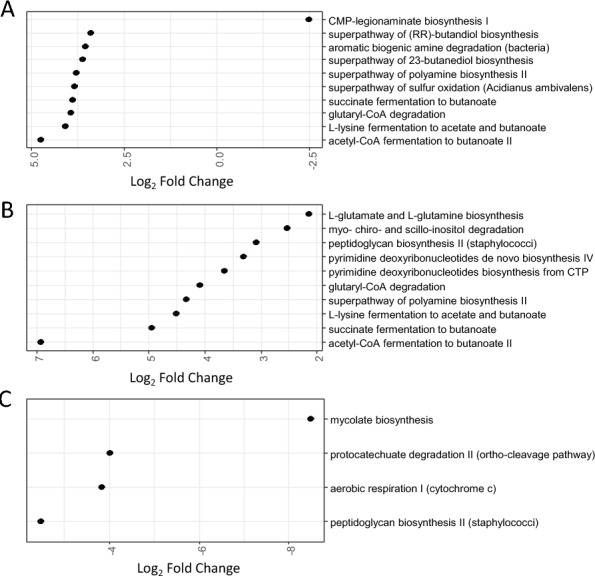
**Metabolic pathways presenting differential enrichment in the fecal samples**. Functional gene families and MetaCyc pathways were predicted on the basis of the taxa abundances using PICRUSt2 on microbiota at days 6 (**A**), 8 (**B**) and 21 (**C**). No differentially enriched pathways were observed at 12 and 14 days of age. Positive values of log_2_ fold changes correspond to pathways enriched in the SE group; reciprocally, negative values of log_2_ fold changes correspond to pathways enriched in the Φ + SE group.

The changes observed in gut microbial composition do not necessarily mean that the functions present in the microbiota have also changed, as several bacterial taxa share the same functions. To determine whether the differences observed could have an impact on the functions carried by the microbiota, the differences between the SE and Φ + SE groups were then assessed at a functional level on the basis of taxon abundances. This assessment was done using PICRUSt2 (Figures 7 and 8). A total of 69 metabolic pathways were differentially enriched between the Φ + SE and SE groups. Most of differences were observed in cecal samples (45 pathways, across all time points) with a notable concentration at 6 days of age, i.e., before *Salmonella* inoculation, where 45 pathways were differentially enriched in both fecal and cecal samples. At 6 days of age in the cecal samples, 28 pathways were significantly enriched in the SE group. These included ten pathways categorized under “Biosynthesis”, ten under “Degradation/Utilization/Assimilation”, six under “Generation of Precursor Metabolites and Energy”, and two belonging to other categories (Figure 7). While these pathways do not share a single, specific function, they collectively contribute to a wide array of metabolic and physiological processes. They can be grouped into six functional categories, as annotated by letters in Figure 7. “C”: carbon utilization and biodegradation, enabling bacteria to use various carbon sources; “A”: amino acid and polyamine metabolism, supporting bacterial survival and stress responses; “N”: nucleotide metabolism and DNA synthesis, which are essential for DNA replication and bacterial growth; and “E”: energy production and fermentation, enabling survival in oxygen-limited environments. This previous category could be related to pathways involved in fermentation and anaerobic respiration grouped in category “R”: electron transport and alternative respiration. The final category, “S”: biosynthesis of cell components and surface structures, is crucial for bacterial structure and immune evasion.

Interestingly, the “peptidoglycan biosynthesis II (staphylococci)” pathway was significantly enriched in the cecal samples at 6, 8, and 12 days of age in the SE group. This enrichment likely reflects the increased presence of Gram-positive bacteria in these samples, as all taxa enriched at 6 DoA in these samples belonged to Gram-positive bacteria. The other two structural pathways can be found in Gram-positive and Gram-negative bacteria [37]. In addition to structural pathways, 13 metabolic pathways related to fermentation, and included in the category “R,” were enriched in the SE group at 6 DoA. These pathways include pyruvate fermentation to acetone, pyruvate fermentation to propanoate I, the superpathway of glycerol degradation to 1,3-propanediol, l-lysine fermentation to acetate and butanoate, the superpathway of *Clostridium acetobutylicum* acidogenic fermentation, pyruvate fermentation to butanoate, succinate fermentation to butanoate, glutaryl-CoA degradation, allantoin degradation IV (anaerobic), isopropanol biosynthesis, the superpathway of 2,3-butanediol biosynthesis, the superpathway of (R,R)-butanediol biosynthesis, and aromatic biogenic amine degradation. An additional pathway related to anaerobic respiration (the superpathway of menaquinol-8 biosynthesis II) was also enriched. This pattern suggests that the microbial community in the SE ceca favors anaerobic metabolism. Indeed, only five pathways that require oxygen were enriched (catechol degradation I, urea cycle, octane oxidation, l-glutamate degradation V, and mannan degradation).

The Φ + SE group showed a markedly different metabolic profile. At 6 DoA, only one metabolic pathway related to fermentation and anaerobic respiration (2-methylcitrate cycle I and II) was enriched. In contrast, three oxygen-requiring pathways related to the tricarboxylic acid (TCA) cycle (l-histidine degradation II, ketogluconate metabolism, and 3-phenylpropanoate degradation) were enriched. The other significantly enriched pathways are active in both aerobic and anaerobic conditions (Figure 7). This pattern suggests that the cecal environment in the Φ + SE group may have been more oxygenated, explaining the presence of oxygen-consuming bacteria.

These metabolic contrasts were less apparent in fecal samples. In the SE group, only four metabolic pathways related to fermentation and three oxygen-requiring pathways were enriched (Figure 8). In the Φ + SE group, only a single oxygen-dependent metabolic pathway, related to protocatechuate degradation II, showed significant enrichment. Most other differentially enriched pathways in the fecal microbiota could function under both oxygen-rich and oxygen-poor conditions (Figure 8), highlighting the benefit of analyzing cecal samples for insights into the microbial metabolic environment.

## Discussion

In the present study, we demonstrated that a recurrent administration of a phage cocktail can significantly influence the course of the gut microbiota development before and after *Salmonella* inoculation. This result is supported by our initial findings, indicating that although the phages in the cocktail primarily targeted *Salmonella*, they also exhibited activity against other *Enterobacteriaceae* such as different *E. coli* strains. Such a broader host range may contribute to the observed shifts in microbial composition. In addition, previous studies have shown that phages can impact the gut microbiota beyond their direct bacterial targets and even in the absence of their natural host [5, 8, 9]. These indirect effects are often mediated through ecological cascades, where alterations in one microbial population indirectly influence others—even those not susceptible to the phages—through mechanisms such as nutrient competition, metabolic cross-feeding, or immune modulation.

This demonstration was based on a characterization of the gut microbiota at two levels, the cecal and the fecal. Ceca are indeed the main site of *Salmonella* colonization in infected chicks, while fecal shedding is important for understanding the transmission of commensal and pathogenic intestinal bacteria between animals and in the environment [38, 39].

Previous studies have shown that the chicken fecal microbiota can be considered has a proxy for the cecal microbiota [40]. However, another study has shown that fecal microbiota has limited potential as a proxy in studies of chicken gut microbial community [41]. Our results showed that α-diversity was systematically lower in feces compared with ceca. Thus, fecal samples corresponded to a fraction of the α-diversity harbored in the ceca. Moreover, we demonstrated, using β-diversity indices and taxon identification, that fecal and cecal samples were qualitatively different. While fecal samples were dominated at the genus level by *Lactobacillus* and *Romboutsia*, most cecal samples included a large fraction of unknown genera from the *Lachnospiraceae* family and of *Enterococcus* and *Ruminococcus* (*torques* group) genera throughout the experiment. Some authors suggested that collected fecal samples only accurately reflect the cecal composition if they are collected after cecal voiding [42]. Here, the fecal samples were collected when awaiting the production of fresh droppings. In this context, they may be close to the ileal microbiota if collected before a cecal voiding cycle. This difference could explain the discrepancy between the studies. Thus, in our condition, the quantitative and qualitative differences between the fecal and cecal samples observed argue for separate analyses of the impact of phage administration.

Comparison of the gut microbiota composition between chicks that received or did not receive phages revealed significant differences in both microbial succession (evolutionary kinetics) and taxonomic composition across the fecal and cecal samples. These differences were unlikely related to a batch effect as both groups (Φ + SE and SE) were placed in the same room, under identical environmental conditions, though physically separated to prevent cross-contamination. These differences were mainly observed during the first few days of age. In the cecal microbiota, α-diversity shifted markedly between 6 and 12 DoA in both groups, while the fecal microbiota showed only modest changes over the same period. At the fecal level, the most important modification corresponded to a sharp increase in *Romboutsia* and a concurrent decline in *Lactobacillus* from 6 to 12 DoA. However, these differences disappeared over time, indicating a potential convergence of the fecal microbiota between groups as the birds aged.

Interestingly, the Φ + SE group exhibited greater temporal variations in both α- and β-diversity, particularly in ceca, suggesting that phage administration modulated the early developmental trajectory of the gut microbiota—most strongly in the cecum, and to a lesser extent in feces (comparison of Figures 5 and 6). This conclusion is further supported by the consistently greater divergence in cecal microbiota composition between the Φ + SE and SE groups across all time points, whereas fecal microbiota differences reached statistical significance at only two time points, with the most substantial divergence occurring at 6 DoA. Since *Salmonella* challenge occurred at 7 DoA, the microbial differences observed at 6 DoA—particularly in the ceca—are likely attributable to the early administration of the phage cocktail. This finding is of particular importance, as the ceca are the crucial sites of *Salmonella* colonization.

Overall, these results suggest that phage treatment not only influences pathogen dynamics but also reshapes the broader gut microbial community during a critical window of early colonization. This modulation, especially within the ceca, continues with time as few OTUs were still differentially abundant in the cecal and fecal samples after infection (i.e., after 7 days of age), confirming that the changes were mainly because of phage treatment rather than *Salmonella* colonization. Moreover, after 12 DoA, the differences in OTUs decreased sharply between the Φ + SE and SE groups, suggesting that animal-to-animal transmission, age, or *Salmonella* colonization should have induced, in the long-term, a homogeneous gut microbial composition in the Φ + SE and SE groups. These results are in agreement with previous studies showing that phage administration did not affect the long-term transition to a complex and homogeneous microbiota [43], even in the context of a *Salmonella* infection. In line with this, and in accordance with our results, Zhao et al. showed that phage administration mainly impacted the early cecal microbiota [5]. The impact of phages on the fecal microbiota was rarely studied; most phage studies are based on cecal [5, 43] or ileal [44] microbiota. An impact of phages on fecal microbiota composition has nevertheless been demonstrated in a murine model [45]. However, our study shows that the potential impact of the phage cocktail presented here, was significant on the cecal microbiota but was limited to a few taxa on the fecal microbiota.

The *Enterobacteriaceae* family was of particular interest here, as some of the phages in the cocktail can also infect (in vitro) certain strains of *E. coli*, which are genetically close to their main target, *Salmonella*. In line with this result, we observed that several taxa assigned to the *Enterobacteriaceae* were depleted in cecal samples from the Φ + SE group at the beginning of the experiment. It may be noted that the taxa considered represented only a negligible fraction of the cecal gut microbiota. However, it has been described that *E. coli* are among *Salmonella*’s best competitors [46, 47], and they may in turn have affected the whole gut microbiota through cascading effects on other taxa in this complex ecosystem, as described previously [9]. Assessing the host range of the phage cocktail would require further tests on a wider range of potential targets.

We therefore observe many compositional differences that seem to be induced by phage treatment. This is particularly true at 6 days of age, i.e., before *Salmonella* inoculation. The study of the putative functions of the cecal microbiota at this age shows that these changes in taxa are concomitant with a change in the ecological niche. Thus, phage-treated animals have a microbiota that can use sugars in the tricarboxylic acid (TCA) cycle via oxygen to produce energy, which is not the case for untreated animals whose microbiota can provide energy mainly via fermentation and anaerobic respiration. This is consistent with a greater abundance of SCFA-producing bacteria in phage-untreated animals. This metabolic shift in the microbiota may have important consequences, as we know the crucial role of fermentation and anaerobic environment in gut health [48] as well as in the control of *Salmonella* colonization [20, 21, 49]. This indirect effect of phage inoculation on microbiota could partly explain the lower efficacy observed in the present experiment compared with the previously described preventive treatment conducted under similar conditions in term of bacteria, chicken line, and *Salmonella* strain [18]. Specifically, in the earlier preventive treatment, phage administration led to a 2.9-log reduction in *Salmonella* levels in ceca, and a 1.9-log reduction in feces, 4 days after infection. In contrast, in the current experiment where phages were inoculated before and after infection, only a 0.4-log reduction in ceca, and a 1.1-log reduction in feces, were observed 5 days after infection. This reduced efficacy following recurrent phage administration could be due to antagonistic effects. Even though administering phages after infection might theoretically improve the elimination of *Salmonella*, this benefit may be counteracted by changes in the intestinal environment. The reduction in anaerobiosis may render the intestinal niche more favorable to *Salmonella* colonization and proliferation, facilitating the outgrowth of *Salmonella* compared with the microbiota. By contrast, the reduction of *Salmonella* colonization observed in our previous study may be caused by a direct action of the phages, without undergoing the effects of subsequent changes to the microbiota.

Overall, the results of this study indicate that recurrent administration of phages transiently affected cecal and the fecal microbiota. Given the total amount of phages inoculated and the potential negative effect on microbiota composition and function we observed, recurrent use of phages should be considered with caution. The design of phage cocktails must therefore take into account not only the efficacy of phages against the targeted pathogens but also their persistence in vivo, the induction of bacterial resistance, and the direct and indirect effects on gut microbiota.

## Data Availability

The raw 16S rRNA gene sequencing data generated and analyzed during the current study are available in the Sequence Read Archive (SRA) of the European Nucleotide Archive (ENA) under the accession numbers ERR10793000 to ERR10793144 of the bioproject PRJEB59053.
